# Kristen rat sarcoma virus ( *KRAS* ) G12F‐positive non‐small cell lung cancer mimicking *KRAS* G12C positivity: A case report

**DOI:** 10.1111/1759-7714.15171

**Published:** 2023-11-27

**Authors:** Issei Oi, Takanori Ito, Zentaro Saito, Takuma Imakita, Osamu Kanai, Kohei Fujita, Hiromasa Tachibana, Tadashi Mio

**Affiliations:** ^1^ Division of Respiratory Medicine, Center of Respiratory Diseases National Hospital Organization Kyoto Medical Center Kyoto Japan

**Keywords:** AMOY, G12C, G12F, KRAS

## Abstract

Searching for driver gene alteration is a prerequisite for chemotherapy of non‐small cell lung cancer. Due to its high sensitivity and concordance rate, the Amoy Dx Pan Lung Cancer PCR panel has been approved and is widely used in Japan. In this report, we describe a case in which a positive result for Kristen rat sarcoma virus (*KRAS*) exon2 p.G12F, a rare *KRAS* mutation, may have led to a false‐positive result for *KRAS* exon2 p.G12C on AMOY. Genetic analysis in this case was performed by LC‐SCRUM‐Asia.

## Graphical abstract

Both *KRAS* G12C and G12F exhibit c34G > T sequencing, but the same site is being evaluated for the presence of *KRAS* G12C mutations in AMOY. As a result, false positives in AMOY for *KRAS* G12C are indicated by *KRAS* G12F.

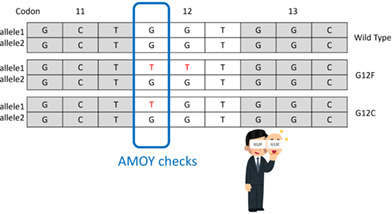

## INTRODUCTION

The search for targetable driver oncogene alteration in non‐small cell lung cancer (NSCLC) is essential for determining appropriate treatment, and testing is recommended prior to treatment.^1^, ^2^, ^3^, ^4^ The Oncomine Dx Target Test Multi‐CDx System and the Amoy Dx Pan Lung Cancer PCR panel are widely used as multiplex tests in Japan, but there are some differences, such as the former is next‐generation sequencing (NGS) and the latter is real‐time polymerase chain reaction (PCR)‐based, as well as in detectable gene mutations and the turnaround time (TAT) from specimen submission to the final test result.

Kristen rat sarcoma virus (*KRAS*) is the most common genetic mutation in NSCLC in the U.S. and Europe, along with epidermal growth factor receptor, and is found in approximately 30% of Western lung adenocarcinomas^5^ and 10%–14% of Japanese lung adenocarcinomas.^6^, ^7^ *KRAS* exon2 p.G12C (*KRAS* G12C) mutation is now an important test target because a molecularly targeted drug called sotorasib has shown efficacy^8^ and been approved for *KRAS* G12C‐positive lung cancer. *KRAS* exon2 p.G12F (*KRAS* G12F), which is also a type of *KRAS* mutation, but is considered a rare genetic mutation, accounting for only 1% of *KRAS*‐positive non‐small cell lung cancer.^9^ In this study, we report a case in which *KRAS* G12C was falsely positive on AMOY which was actually showing *KRAS* G12F positive.

## CASE REPORT

**FIGURE 1:**
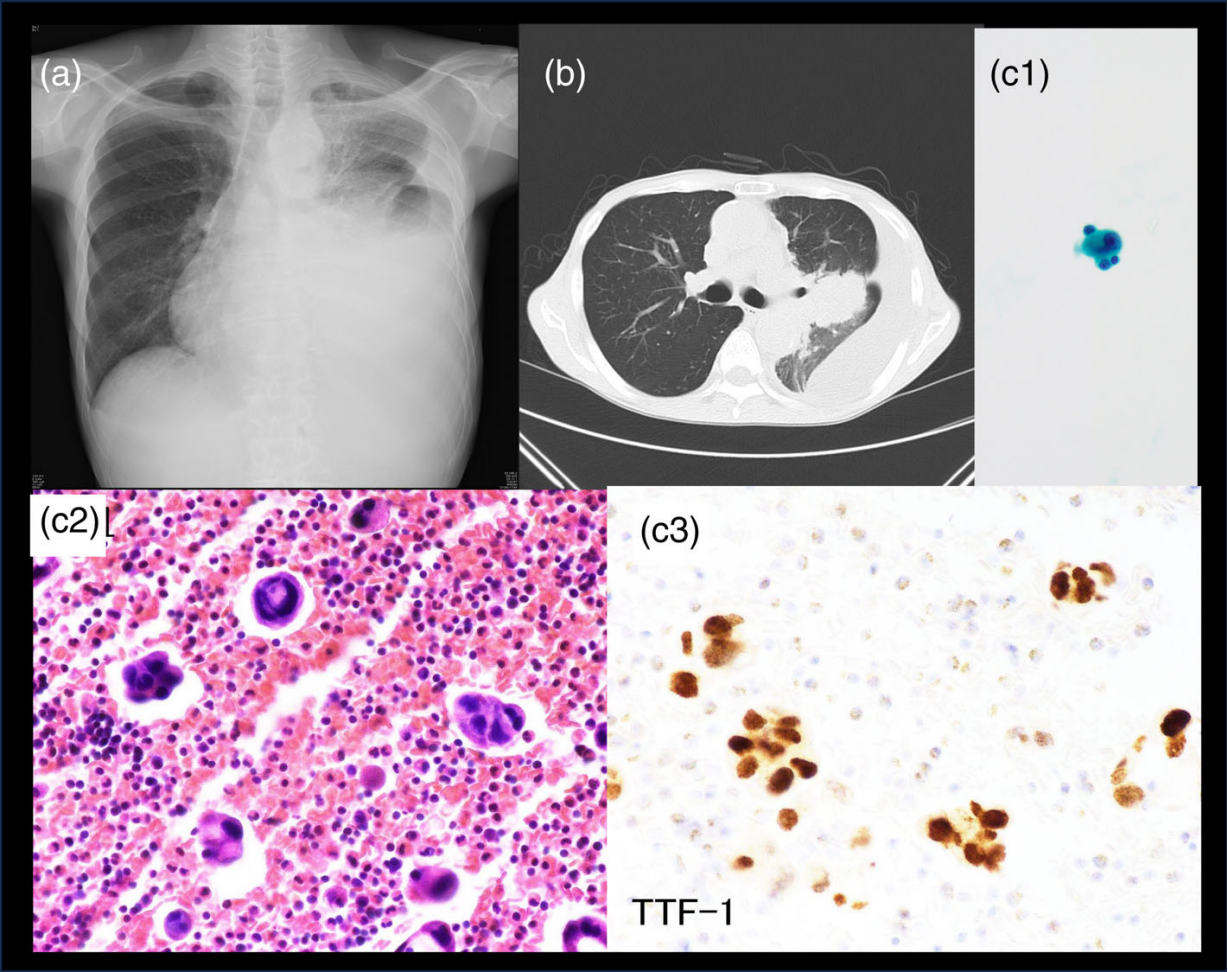
Radiological findings and cytology. The patient had left pleural effusion, and a tumor was found in the left upper lobe. Cytology of pleural fluid revealed malignant cells and thyroid transcription factor‐1 (TTF‐1) stain of the cell block was positive, indicating adenocarcinoma. (a) Chest X‐ray. (b) Chest computed tomography (CT) scan. (c1) Cytology. (c2) Hematoxylin–eosin staining of cell block prepared from pleural effusion, ×400. (c3) TTF‐1 staining of cell block prepared from pleural effusion, ×400.

A 67‐year‐old male presented with dyspnea and was referred to our institution after the discovery of right pleural effusion during a previous medical examination (Figure 1A,B). Thoracentesis was performed, and cytology revealed BerEP4 positivity, thyroid transcription factor‐1 positivity and adenocarcinoma of the lung in the pleural fluid cell block (Figure 1C1‐3). Pleural fluid was submitted to LC‐SCRUM‐Asia, where both AMOY and Oncomine Precision Assy (Oncomine) (Thermo Fisher Scientific) testing were performed. LC‐SCRUM‐Asia, previously named LC‐SCRUM‐Japan, is a prospective, nationwide, clinical, and genomic screening program for lung cancer (UMIN ID: UMIN000010234). AMOY showed positivity for all *KRAS* G12C, G12D/S, and G12A/V/R, G13C mutations, while Oncomine indicated positivity for *KRAS* exon 2 p.G12D (*KRAS* G12D) and G12F mutations. The therascreen *KRAS* PCR kit (Qiagen) confirmed the absence of the G12C mutation. The false‐positive G12C result in AMOY due to the presence of G12F positivity was diagnosed. The patient had spinal canal extension of bone metastases, for which radiation therapy was initiated prior to starting chemotherapy with pembrolizumab.

## DISCUSSION

AMOY is a PCR method that measures the presence or absence of each gene mutation by observing the nucleotide sequence. AMOY has an excellent sensitivity and short TAT,^10^ and is currently approved in Japan as a companion diagnostic function for advanced stage lung cancer. The corresponding c.34G > T sequence is used to confirm the presence or absence of *KRAS* G12C in AMOY. However, *KRAS* G12F has also been found to exhibit c.34G > T,^9^ although it is not subject to AMOY detection. The amino acid substitutions and corresponding base substitutions in *KRAS* G12 are shown in Table 1.^11^ *KRAS* G12F also has c.35G > T,^9^ but it is not subject to detection, leading to a false positive result for *KRAS* G12C in AMOY. Furthermore, the coexistence of C.34G > T and c.35G > T was reported in 6% of c.34G > T cases, 70% of which were G12F, but other patterns such as concomitant G12C and G12V or G12C and G12F were also observed;^9^, ^12^ therefore caution is required in interpreting the results of this study because only one of the panels can be used under the Japanese insurance system. Fortunately, the design of the LC‐SCRUM‐Asia study allowed for simultaneous testing of Oncomine and AMOY in the present case, and thus we could confirm a positive result for G12F and a negative result for G12C. It is desirable to take necessary measures such as the addition of a therascreen that detects G12C with GGT > TGT (c.34G > T) as appropriate. Additionally, the simultaneous mutation of G12D (c.35G > A) and G12F detected in this study has not been previously reported,^9^, ^12^, ^13^, ^14^ and is considered to be a very rare duplication. It has been suggested that when *KRAS* G12C and G12F coexist, G12C may be the preceding genetic mutation,^9^ but the clinical significance of the mutation demonstrated in this case is unknown.

**TABLE 1:** The pattern of substitution in *KRAS* G12‐positive lung cancer.

*KRAS* G12 amino acid substitution	Base substitution		
	c.34G>	c.35G>	c.36T>
G12C	T	G	T
	T	G	C
G12V	G	T	T
	G	T	C
	G	T	G
G12D	G	A	T
G12A	G	C	T
	G	C	A
G12S	A	G	T
	T	C	T
G12R	C	G	T
	A	G	A
G12F	T	T	T
G12L	C	T	T
	C	T	G
G12I	A	T	T
G12W	T	G	G
G12E	G	A	A
	G	A	G
G12Y	T	A	T
G12N	A	A	T
G12H	C	A	T

Abbreviations: A, adenine; C, cytosine; G, guanine; *KRAS*, Kristen rat sarcoma virus; T, thymine.

## AUTHOR CONTRIBUTION

Issei Oi: Conceptualization, Methodology, Writing‐original draft, Supervision, Project administration Takanori Ito, Zentaro Saito, Takuma Imakita, Osamu Kanai, Kohei Fujita, and Hiromasa Tachibana: Case Management, Writing‐original draft, Investigation. Tadashi Mio: Funding acquisition, Supervision.

## CONFLICT OF INTEREST STATEMENT

The authors declare no conflict of interest.

## References

[1] Planchard D , Popat S , Kerr K , Novello S , Smit EF , Faivre‐Finn C , et al. Metastatic non‐small cell lung cancer: ESMO Clinical Practice Guidelines for diagnosis, treatment and follow‐up. Ann Oncol. 2018;29:iv192–iv237. 10.1093/annonc/mdy275 30285222

[2] Kalemkerian GP , Narula N , Kennedy EB , Biermann WA , Donington J , Leighl NB , et al. Molecular testing guideline for the selection of patients with lung cancer for treatment with targeted tyrosine kinase inhibitors: American Society of Clinical Oncology endorsement of the College of American Pathologists/International Association for the Study of Lung Cancer/Association for Molecular Pathology Clinical Practice Guideline Update. J Clin Oncol. 2018;36(9):911–919. Available from: 10.1200/JCO.2017.76.7293 29401004

[3] Ettinger DS , Wood DE , Aisner DL , Akerley W , Bauman JR , Bharat A , et al. Non–small cell lung cancer, version 3.2022, NCCN Clinical Practice Guidelines in oncology. J Natl Compr Cancer Netw. 2022;20(5):497–530. Available from: https://jnccn.org/view/journals/jnccn/20/5/article-p497.xml 10.6004/jnccn.2022.0025 35545176

[4] Howlader N , Forjaz G , Mooradian MJ , Meza R , Kong CY , Cronin KA , et al. The effect of advances in lung‐cancer treatment on population mortality. N Engl J Med. 2020;383(7):640–649. 10.1056/NEJMoa1916623 32786189 PMC8577315

[5] Prior IA , Hood FE , Hartley JL . The frequency of Ras mutations in cancer. Cancer Res. 2020;80(14):2969–2974. Available from: 10.1158/0008-5472.CAN-19-3682 32209560 PMC7367715

[6] Saito M , Shiraishi K , Kunitoh H , Takenoshita S , Yokota J , Kohno T . Gene aberrations for precision medicine against lung adenocarcinoma. Cancer Sci. 2016;107(6):713–720. Available from: 10.1111/cas.12941 27027665 PMC4968599

[7] Tamiya Y , Matsumoto S , Zenke Y , Yoh K , Ikeda T , Shibata Y , et al. Large‐scale clinico‐genomic profile of non‐small cell lung cancer with KRAS G12C: Results from LC‐SCRUM‐Asia study. Lung Cancer. 2023;176:103–111. 10.1016/j.lungcan.2022.12.019 36634571

[8] Skoulidis F , Li BT , Dy GK , Price TJ , Falchook GS , Wolf J , et al. Sotorasib for lung cancers with KRAS p.G12C mutation. N Engl J Med. 2021;384(25):2371–2381. 10.1056/NEJMoa2103695 34096690 PMC9116274

[9] Vaclova T , Chakraborty A , Sherwood J , Ross S , Carroll D , Barrett JC , et al. Concomitant KRAS mutations attenuate sensitivity of non‐small cell lung cancer cells to KRAS G12C inhibition. Sci Rep. 2022;12(1):2699. 10.1038/s41598-022-06369-3 35177674 PMC8854729

[10] Kunimasa K , Matsumoto S , Kawamura T , Inoue T , Tamiya M , Kanzaki R , et al. Clinical application of the AMOY 9‐in‐1 panel to lung cancer patients. Lung Cancer. 2023;179:107190. 10.1016/j.lungcan.2023.107190 37058787

[11] COSMIC . ver 98. Available from: https://cancer.sanger.ac.uk/cosmic

[12] Campbell JD , Alexandrov A , Kim J , Wala J , Berger AH , Pedamallu CS , et al. Distinct patterns of somatic genome alterations in lung adenocarcinomas and squamous cell carcinomas. Nat Genet. 2016;48(6):607–616. 10.1038/ng.3564 27158780 PMC4884143

[13] Hoadley KA , Yau C , Hinoue T , Wolf DM , Lazar AJ , Drill E , et al. Cell‐of‐origin patterns dominate the molecular classification of 10,000 tumors from 33 types of cancer. Cell. 2018;173(2):291–304. 10.1016/j.cell.2018.03.022 29625048 PMC5957518

[14] Jordan EJ , Kim HR , Arcila ME , Barron D , Chakravarty D , Gao J , et al. Prospective comprehensive molecular characterization of lung adenocarcinomas for efficient patient matching to approved and emerging therapies. Cancer Discov. 2017;7(6):596–609. Available from: 10.1158/2159-8290.CD-16-1337 28336552 PMC5482929

